# Increased Risk of Migraine in Patients with Temporomandibular Disorder: A Longitudinal Follow-Up Study Using a National Health Screening Cohort

**DOI:** 10.3390/diagnostics10090724

**Published:** 2020-09-20

**Authors:** Soo-Hwan Byun, Chanyang Min, Dae-Myoung Yoo, Byoung-Eun Yang, Hyo-Geun Choi

**Affiliations:** 1 Department of Oral & Maxillofacial Surgery, Dentistry, Hallym University College of Medicine, Anyang, Gyeonggi-do 14068, Korea; purheit@daum.net (S.-H.B.); face@hallym.ac.kr (B.-E.Y.); 2 Research Center of Clinical Dentistry, Hallym University Clinical Dentistry Graduate School, Chuncheon, Gangwon-do 24252, Korea; 3 Hallym Data Science Laboratory, Hallym University College of Medicine, Anyang, Gyeonggi-do 14068, Korea; joicemin@naver.com (C.M.); ydm1285@naver.com (D.-M.Y.); 4 Department of Otorhinolaryngology-Head & Neck Surgery, Hallym University College of Medicine, Anyang, Gyeonggi-do 14068, Korea

**Keywords:** migraine, TMD, Korean National Health Insurance Service, cohort, aura

## Abstract

Background: The aim of this study was to investigate the association between temporomandibular disorder (TMD) and migraine through a longitudinal follow-up study using population data from a national health screening cohort. Methods: This cohort study used data from the Korean National Health Insurance Service-Health Screening Cohort from 2002 to 2015. Of the 514,866 participants, 3884 TMD patients were matched at a 1:4 ratio with 15,536 control participants. Crude models and models adjusted for obesity, smoking, alcohol consumption, systolic blood pressure, diastolic blood pressure, fasting blood glucose, total cholesterol, and Charlson Comorbidity Index (CCI) scores were calculated. Chi-squared test, Kaplan–Meier analysis, and two-tailed log-rank test were used for statistical analysis. Stratified Cox proportional hazard models were used to assess hazard ratios (HR) and 95% confidence intervals (CIs) for migraine in both control groups. Results: The adjusted HR for migraine was 2.10 (95% CI: 1.81–2.44) in the TMD group compared to the control group, which was consistent in subgroup analyses according to age, sex, and Kaplan–Meier analysis. Conclusions: This study demonstrated that TMD patients have a higher risk of migraine. These results suggest that dentists can decrease the risk of migraine in TMD patients by managing TMD properly.

## 1. Introduction

Temporomandibular disorder (TMD) is a collective term for comprehensive clinical symptoms related to the dysfunction of the temporomandibular joint (TMJ), masticatory muscles, and adjacent anatomic structures [1]. The etiology of TMD is multifactorial, including parafunctional habit, posture, and neurologic factors [2,3]. Mastication and other functions aggravate the condition, and most patients suffer from limited or asymmetric mouth opening. Related symptoms of TMD are headache, joint sounds (clicking, popping, and crepitus), and craniomaxillofacial pain [4]. TMD affects between 5–70% of Caucasians, and several studies have reported that maxillofacial pain is the major complaint of more than half of the consultations and up to 80% of dental appointments among adolescents [5,6]. Moreover, it was shown that clinicians feel incompetent in managing TMD, resulting in referrals to other clinicians [7]. 

Migraine usually occurs on one side of the head with throbbing pain or a pulsing sensation. The symptoms often occur with photosensitivity, vomiting, and nausea. Migraine can last for several hours, and it can interfere with normal activities. Medications could relieve some migraines and prevent them. Proper medications, combined with self-help solutions and healthier lifestyles, might help to manage this headache [8].

The International Classification of Headache Disorders (ICHD) has classified migraine into two types: with and without aura [9]. Based on the classification of ICHD-3, an aura must present with at least three of the following six symptoms: spreading gradually for more than 5 min, two or more symptoms occurring in succession, each individual aura symptom lasts 5–60 min, at least one aura symptom is unilateral, at least one aura symptom is positive, and the aura is accompanied or followed within 60 min by headache [9,10]. An aura is known as a warning sign prior to migraine for some patients. An aura can occur with visual disturbances, including blind spots, flashes of light, tingling on one side of the face, or difficulty speaking. Migraine with aura is considered to affect between one-fifth and one-third of those with migraine in the United States, an estimated 7.4–11.1 million people [11]. The pathophysiology of aura is widely known as cortical spreading depression (CSD) [12]. CSD is activated by slow depolarization in cortical neurons and glia, followed by hyperpolarization that moves across the cortex at a rate of 3–5 mm/min. It is accompanied by alterations in neurotransmitter release and ion homeostasis [13]. As greater energy is needed to restore homeostasis, this is accompanied by a rapid spike in cerebral blood flow [14].

A migraine without aura is the most common type of migraine, comprising approximately 75% of all migraines [9]. This type of migraine develops without aura, but it can present with various symptoms at its initial stages. According to ICHD-3, it lasts for 4–72 h and has at least two of the following headache characteristics: moderate-to-severe intensity, unilateral location, aggravation by physical activity, and pulsating quality [15]. One or more associated symptoms such as nausea/vomiting and photophobia/phonophobia would happen during the attack. In addition, attacks of a migraine without aura must not be attributable to another disorder.

Most painful symptoms are transient and are related to a specific lesion or disease that can be cured. Unfortunately, some types of pain are chronic, and chronic pain remains a public health issue [16]. Both TMD and migraine could be main causes of chronic pain in the orofacial area. Many patients with TMD have several comorbid conditions [17,18]. Moreover, previous studies of TMD patients have revealed that comorbid conditions are the reason for 50% of TMD patients requiring care for TMD symptoms, and for 20% of patients with long-term disability from their pain [19,20,21]. It is essential that any comorbid conditions and their influences on clinical outcomes are identified and evaluated by clinicians managing TMD patients [22].

Some studies have reported an association between TMD and migraine [8,23,24,25,26]. This association was thought to be induced by anatomic, neurologic, and emotional relationships. Previous studies reported that migraine is related to pain in the sinus, teeth, TMJ, and cervical areas [27,28,29]. However, most studies have been based on limited participants or subjective questionnaires [26,30].

The aim of this study was to investigate the association between TMD and migraine by conducting a longitudinal study using population data from a national health screening cohort. It was determined that patients with TMD have a greater risk of migraine than those without TMD.

## 2. Materials and Methods

### 2.1. Study Population

The ethics committee of Hallym University approved this study on 4 November 2019 (No. 2019-10-023). The need for written informed consent was waived by the Institutional Review Board. All analyses adhered to the guidelines and regulations of the ethics committee. The details of the Korean National Health Insurance Service-Health Screening Cohort data have been described elsewhere [31].

### 2.2. Definition of Temporomandibular Disorder

TMD was defined if participants were diagnosed with the ICD (International Classification of Diseases)-10 code K07.6 (Temporomandibular joint disorders). For diagnostic accuracy, this study only selected participants who were treated ≥2 times for the diagnosis of TMD.

### 2.3. Definition of Migraine

Migraine was defined if participants were diagnosed with the ICD-10 code G43 (Migraine). For diagnostic accuracy, this study only selected participants who were treated ≥2 times for the diagnosis of migraine. Among them, migraine with aura was defined if participants were diagnosed with the ICD-10 code G43.1 (Migraine with aura).

### 2.4. Participant Selection

**Figure 1:**
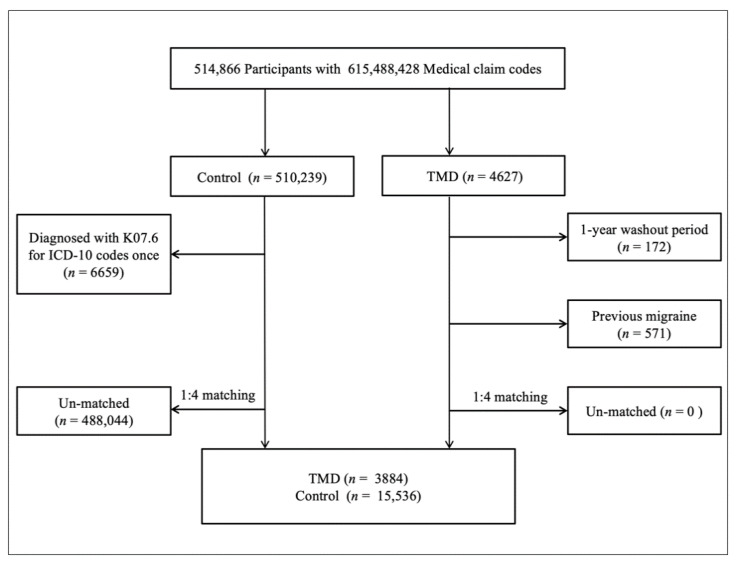
A schematic illustration of the participant selection process. Out of 514,866 participants, 3884 patients with temporomandibular disorder were matched at a 1:4 ratio with 15,536 control participants for age, sex, income, and region of residence. TMD, temporomandibular disorder; ICD-10, International Classification of Diseases, 10th edition.

TMD patients were selected from 514,866 participants with 615,488,428 medical claim codes from 2002 to 2015 (*n* = 4627). The control group consisted of participants who were not diagnosed with TMD from 2002 to 2015 (*n* = 510,239). TMD patients were excluded if they had a 1-year washout period (*n* = 172). Control participants were excluded if they were diagnosed with the ICD-10 code K07.6 once (*n* = 6659). TMD patients were matched at a 1:4 ratio with control participants for age, sex, income, and region of residence; this was done randomly to prevent selection bias. In this study, we supposed that the matched participants were involved in the same date (index date). Participants who died before the index date and had a history of migraine before the index date were excluded. In the TMD group, 571 participants were excluded, and during matching, 488,044 control participants were excluded. As a result, 3884 TMD patients were matched at a 1:4 ratio with 15,536 control participants (Figure 1).

### 2.5. Covariates

Age was categorized into ten groups ranging from 40–44 to 85+. Income groups were divided into five classes from lowest income (class 1) to highest (class 5) income. Regions of residence were grouped into urban and rural areas following our previous study [31].

Tobacco smoking, alcohol consumption, obesity based on body mass index (BMI, kg/m^2^) [32,33], systolic blood pressure (BP), diastolic BP, fasting blood glucose, and total cholesterol were measured as described in our previous study [34]. The Charlson Comorbidity Index (CCI) was used to measure 17 comorbidities [35].

### 2.6. Statistical Analyses

Chi-squared tests were used to compare general characteristics between the TMD and control groups.

Stratified Cox proportional hazard models were used to assess the hazard ratios (HRs) and 95% confidence intervals (CIs) for migraine in the TMD group compared to the control group. In this analysis, crude (simple) and adjusted (for obesity, smoking, alcohol consumption, systolic BP, diastolic BP, fasting blood glucose, total cholesterol, and CCI scores) models were used. Age, sex, income, and region of residence were stratified. Additionally, this study calculated HRs with 95% CIs for migraine with and without aura in the TMD group compared to the control group.

A Kaplan–Meier analysis and the log-rank test were used to analyze the cumulative probability of migraine in the TMD group compared to the control group.

For subgroup analyses, this study divided participants by age and sex (<60 years old and ≥60 years old; males and females) and analyzed the crude and adjusted models. We additionally performed subgroup analyses of crude and adjusted HRs for migraine with and without aura in the TMD group compared to the control group (Tables S1 and S2).

Two-tailed analyses were performed, and significance was defined as *p*-values less than 0.05. SAS version 9.4 (SAS Institute, Cary, NC, USA) was used for statistical analyses.

## 3. Results

The general characteristics for age, sex, income, and region of residence were identical due to matching between the groups (Table 1), while those for obesity, smoking, alcohol consumption, BP, fasting blood glucose, total cholesterol, and CCI were different.

**Figure 2:**
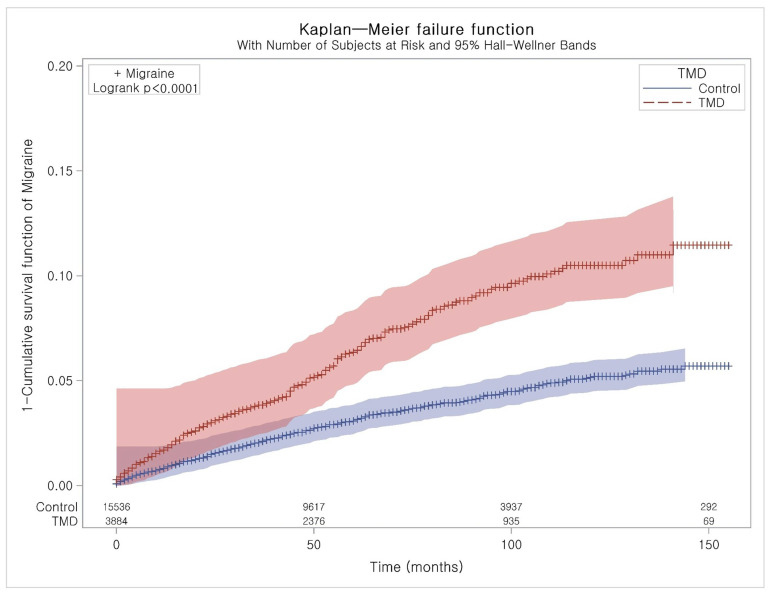
Kaplan–Meier curve of temporomandibular disorder with migraine with and without aura.

The adjusted HR for migraine was 2.10 (95% CI: 1.81–2.44) in the TMD group compared to the control group (Table 2). The results were consistent in subgroup analyses according to age and sex. These were also exhibited in the Kaplan–Meier analysis (Figure 2).

This study additionally analyzed the HRs for migraine with and without aura. The adjusted HR for migraine with aura did not reach statistical significance (Figure S1, Table S1). However, the adjusted HR for migraine without aura was significant in every subgroup (Figure S2, Table S2).

## 4. Discussion

Marklund et al. reported that subjects with TMD had a three-fold greater risk of developing frequent headaches during the 2-year longitudinal study. However, this study did not include a large population [36]. Lim et al. showed that subjects who developed TMD had more headaches compared with those who did not develop TMD and collected data by using a questionnaire [37].

The present study evaluated the association between TMD and migraine by calculating the adjusted HR of migraine after a diagnosis of TMD and used a large population-based dataset which was collected by dentists and physicians who performed objective examinations. The adjusted, statistically significant HR for migraine was 2.10 in the TMD group compared to the control group (*p* < 0.001). The results were consistent in subgroup analyses according to age and sex. These were also shown in the Kaplan–Meier analysis. These results demonstrated that the presence of TMD could increase the risk of migraine. The adjusted HR for migraine with aura did not reach statistical significance (*p* > 0.05). However, the adjusted HR for migraine without aura was significant in every subgroup (*p* < 0.001). These supplementary results could be due to an inaccurate statistical analysis. The association between TMD and migraine is known as a bidirectional link. Both diseases could induce the development of craniomaxillofacial allodynia during painful aggravation. This symptom is associated with peripheral and central sensitization. TMD could activate central sensitization and reduce the pain threshold in migraine [38]. In addition, parafunctional habits and associated painful TMD also could increase the risk for chronic migraine [39,40].

These diseases are related to the common nociceptive system. The preliminary neurons involved in migraine are linked to the first branch of the trigeminal nerve and to the trigeminocervical complex, and those involved in TMD are linked to the neurons of the third branches of the trigeminal nerve [24,41,42]. This nociceptive information converges toward the caudal nucleus of the trigeminal nerve, and from there the pathways of headache and TMD share specific central pathways involved in pain modulation, including the limbic system, brainstem nuclei, sensitive cortex, and thalamus [24]. Neurons in the trigeminal nucleus caudalis combine nociceptive input from intracranial and extracranial tissues and receive supraspinal facilitatory and inhibitory inputs [43]. The neurons integrate all these inputs, transmit the net results to the thalamus, and on to the cortex. Through this convergent point, migraine and TMD may influence each other [23].

Both conditions could share a similar genetic and hormonal basis. A previous study suggested that the association between TMD pain and migraine in women may be partially due to a modest shared genetic risk for both diseases [44]. Sex hormones, such as estrogen, may also control trigeminal nerve sensitization by modulating nociceptive mediators, such as calcitonin gene-related peptide (CGRP) [45]. The OPPERA (Orofacial Pain: Prospective Evaluation and Risk Assessment) study found a complex pattern of considerable changes in biopsychosocial function associated with changes in TMD status. Several biopsychosocial parameters improved among participants with chronic TMD despite pain persisting for years, suggesting considerable potential for ongoing coping and adaptation in response to persistent pain. These biopsychosocial factors could also influence the occurrence of migraine and mutual interaction between TMD and migraine [46].

Based on the results of the present study, clinicians could consider the possibility of improvement in migraine by the treatment of TMD. A few studies have suggested TMD treatment as a solution for migraine. Wright et al. reported that the headache disability score decreased by 17%, the consumption of analgesics was reduced by 18%, and headaches were reduced by 19%, with statistically significant differences, after TMD treatment [47]. Lim et al. showed that the treatment of TMD can improve frequent tension-type headaches associated with TMD secondary to problems of the TMJ [48].

This study had some advantages. First, the data were collected by trained and experienced dentists and physicians. Many previous studies were performed by researchers with questionnaires rather than clinicians [25,30,37,49]. Second, this study utilized a large population-based dataset, the Korean National Health Insurance Service-Health Screening Cohort, which was representative of the Korean population. There have been some studies about the association between TMD and migraine, but most of them were based on data from small populations [23,25,26,30,50]. Moreover, TMD participants were followed up for a maximum of 13 years. Third, various influential factors were adjusted to reduce surveillance bias. This study included multiple confounding factors, such as smoking, alcohol consumption, obesity, and hypertension. Lastly, both TMD and migraine are common conditions, so this study would have great clinical significance for clinicians.

This study also had some disadvantages. First, there were lower numbers of participants for subgroups through the matching procedure. Even though this study started with 514,866 participants, there were only 41 participants with migraine with aura. This may have led to inaccurate results in subgroup analyses. Second, we attempted to adjust for as many factors as possible. However, it was difficult to adjust for all factors, as not all factors were included in the dataset. Finally, the diagnosis of TMD was based on ICD-10. However, to provide the TMD phenotype of a patient population, more accurate criteria such as diagnostic criteria for temporomandibular disorders (DC/TMD) could be utilized. If the diagnosis was made by using standardized and validated criteria such as DC/TMD, the results of this study would be more trustworthy [51].

## 5. Conclusions

This study demonstrated that TMD patients have a higher risk of migraine. This suggests that dentists can decrease the risk of migraine in TMD patients by managing this condition properly.

However, this study did not show that all migraines could be prevented or treated by TMD treatment alone. This study simply showed that TMD could be an influential factor on migraine, so clinicians should be aware of the presence of TMD in migraine patients. If TMD symptoms are found in migraine patients, these symptoms must be managed. In addition, dentists should also determine the presence of migraine in TMD patients. If migraine is confirmed, patients should be referred to the neurology department for further evaluation and treatment.

## Figures and Tables

**Table 1:** General characteristics of participants.

Characteristics	Total Participants		
	TMD (*n*, %)	Control (*n*, %)	*p*-Value
Age (years old)			1.000
40–44	128 (3.3)	512 (3.3)	
45–49	403 (10.4)	1612 (10.4)	
50–54	626 (16.1)	2504 (16.1)	
55–59	629 (16.2)	2516 (16.2)	
60–64	538 (13.9)	2152 (13.9)	
65–69	595 (15.3)	2380 (15.3)	
70–74	512 (13.2)	2048 (13.2)	
75–79	319 (8.2)	1276 (8.2)	
80–84	107 (2.8)	428 (2.8)	
85+	27 (0.7)	108 (0.7)	
Sex			1.000
Male	1753 (45.1)	7012 (45.1)	
Female	2131 (54.9)	8524 (54.9)	
Income			1.000
1 (lowest)	598 (15.4)	2392 (15.4)	
2	505 (13.0)	2020 (13.0)	
3	626 (16.1)	2504 (16.1)	
4	800 (20.6)	3200 (20.6)	
5 (highest)	1355 (34.9)	5420 (34.9)	
Region of residence			1.000
Urban	1908 (40.1)	7632 (40.1)	
Rural	2850 (59.9)	11,400 (59.9)	
Obesity ^†^			
Underweight	112 (2.9)	385 (2.5)	<0.001 *
Normal	1530 (39.4)	5601 (36.1)	
Overweight	1104 (28.4)	4171 (26.9)	
Obese I	1056 (27.2)	4885 (31.4)	
Obese II	82 (2.1)	494 (3.2)	
Smoking status			<0.001 *
Non-smoker	2923 (75.3)	11443 (73.7)	
Past smoker	485 (12.5)	1738 (11.2)	
Current smoker	476 (12.3)	2355 (15.2)	
Alcohol consumption			0.754
<1 time a week	2733 (70.4)	10,892 (70.1)	
≥1 time a week	1151 (29.6)	4644 (29.9)	
Systolic blood pressure			<0.001 *
<120 mmHg	1292 (33.3)	4704 (30.3)	
120–139 mmHg	1882 (48.5)	7508 (48.3)	
≥140 mmHg	710 (18.3)	3324 (21.4)	
Diastolic blood pressure			<0.001 *
<80 mmHg	1964 (50.6)	7306 (47.0)	
80–89 mmHg	1355 (34.9)	5540 (35.7)	
≥90 mmHg	565 (14.6)	2690 (17.3)	
Fasting blood glucose			0.001 *
<100 mg/dL	2540 (65.4)	9787 (63.0)	
100–125 mg/dL	1044 (26.9)	4297 (27.7)	
≥126 mg/dL	300 (7.7)	1452 (9.4)	
Total cholesterol			0.097
<200 mg/dL	2108 (54.3)	8288 (53.4)	
200–239 mg/dL	1294 (33.3)	5115 (32.9)	
≥240 mg/dL	482 (12.4)	2133 (13.7)	
CCI score			0.138
0	2630 (67.7)	10,594 (68.2)	
1	582 (15.0)	2254 (14.5)	
2	337 (8.7)	1206 (7.8)	
3	149 (3.8)	633 (4.1)	
≥4	186 (4.8)	849 (5.5)	
Migraine with/without aura	263 (6.8)	507 (3.3)	<0.001 *
Migraine without aura	253 (6.5)	476 (3.1)	<0.001 *
Migraine with aura	10 (0.3)	31 (0.2)	0.482

CCI, Charlson Comorbidity Index; TMD, temporomandibular disorder. * Chi-squared test, significance at *p* < 0.05. ^†^ Obesity (body mass index, kg/m^2^) was categorized as underweight (<18.5), normal (≥18.5 to <23), overweight (≥23 to <25), obese I (≥25 to <30), or obese II (≥30).

**Table 2:** Crude and adjusted hazard ratios (95% confidence interval) for migraine in temporomandibular disorder and control groups.

Characteristics	Hazard Ratios for Migraine			
	Crude ^†^	*p*-Value	Adjusted ^†,‡^	*p*-Value
Total participants (*n* = 19,420)				
TMD	2.12 (1.83–2.46)	<0.001 *	2.10 (1.81–2.44)	<0.001 *
Control	1.00		1.00	
Age < 60 years old, men (*n* = 4040)				
TMD	2.07 (1.34–3.19)	0.001 *	2.03 (1.31–3.14)	0.002 *
Control	1.00		1.00	
Age < 60 years old, women (*n* = 4890)				
TMD	1.92 (1.49–2.48)	<0.001 *	1.88 (1.46–2.44)	<0.001 *
Control	1.00		1.00	
Age ≥ 60 years old, men (*n* = 4725)				
TMD	2.24 (1.55–3.22)	<0.001 *	2.29 (1.58–3.31)	<0.001 *
Control	1.00		1.00	
Age ≥ 60 years old, women (*n* = 5765)				
TMD	2.30 (1.80–2.93)	<0.001 *	2.28 (1.78–2.91)	<0.001 *
Control	1.00		1.00	

CCI, Charlson Comorbidity Index; TMD, temporomandibular disorder. * Stratified Cox proportional hazard regression model, significance at *p* < 0.05. ^†^ Models were stratified by age, sex, income, and region of residence. ^‡^ The model was adjusted for obesity, smoking, alcohol consumption, systolic blood pressure, diastolic blood pressure, fasting blood glucose, total cholesterol, and CCI scores.

## Supplementary Materials

The following are available online at https://www.mdpi.com/2075-4418/10/9/724/s1, Figure S1: Subgroup analyses of crude and adjusted hazard ratios (95% confidence interval) for migraine with aura in temporomandibular disorder and control groups, Figure S2: Subgroup analyses of crude and adjusted hazard ratios (95% confidence interval) for migraine without aura in temporomandibular disorder and control groups, Table S1: Subgroup analyses of crude and adjusted hazard ratios (95% confidence interval) for migraine with aura in temporomandibular disorder and control groups, Table S2: Subgroup analyses of crude and adjusted hazard ratios (95% confidence interval) for migraine without aura in temporomandibular disorder and control groups.
