# Supplementary material for: Increased Risk of Migraine in Patients with Temporomandibular Disorder: A Longitudinal Follow-Up Study Using a National Health Screening Cohort
Source: Diagnostics (Basel). 2020 Sep 20;10(9):724. doi: 10.3390/diagnostics10090724 (PMC7554700; doi:10.3390/diagnostics10090724)
Supplement: Supplementary file 1 [file diagnostics-10-00724-s001.pdf]

Supplementary Materials

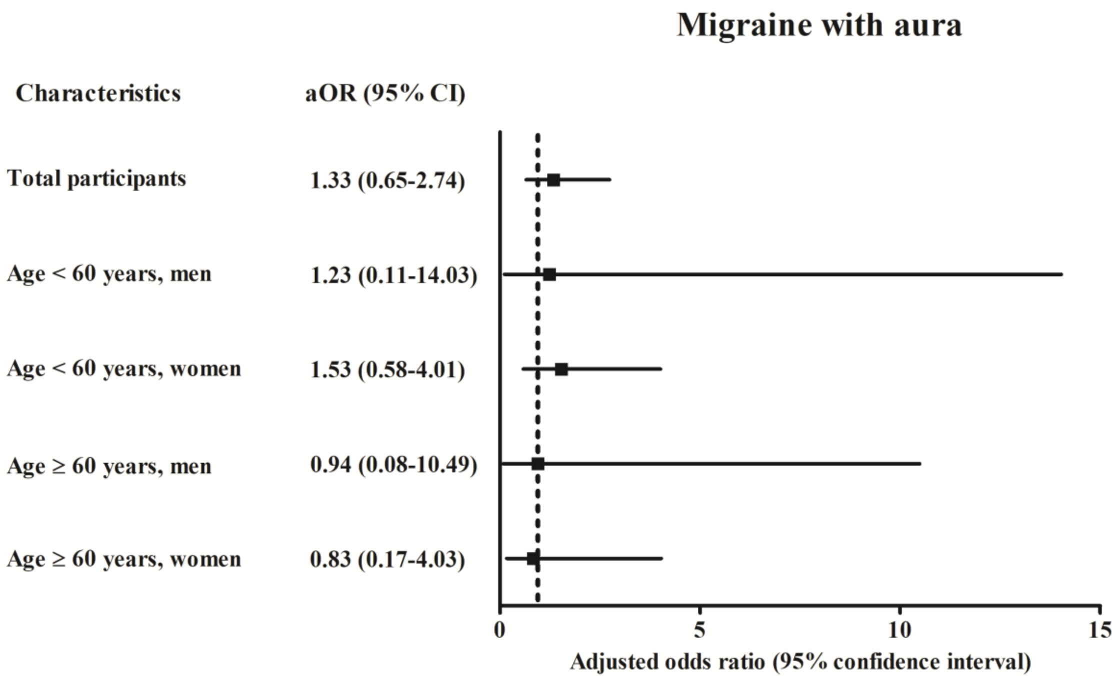

Figure 1. Subgroup analyses of crude and adjusted hazard ratios (95% confidence interval) for migraine with aura in temporomandibular disorder and control groups.

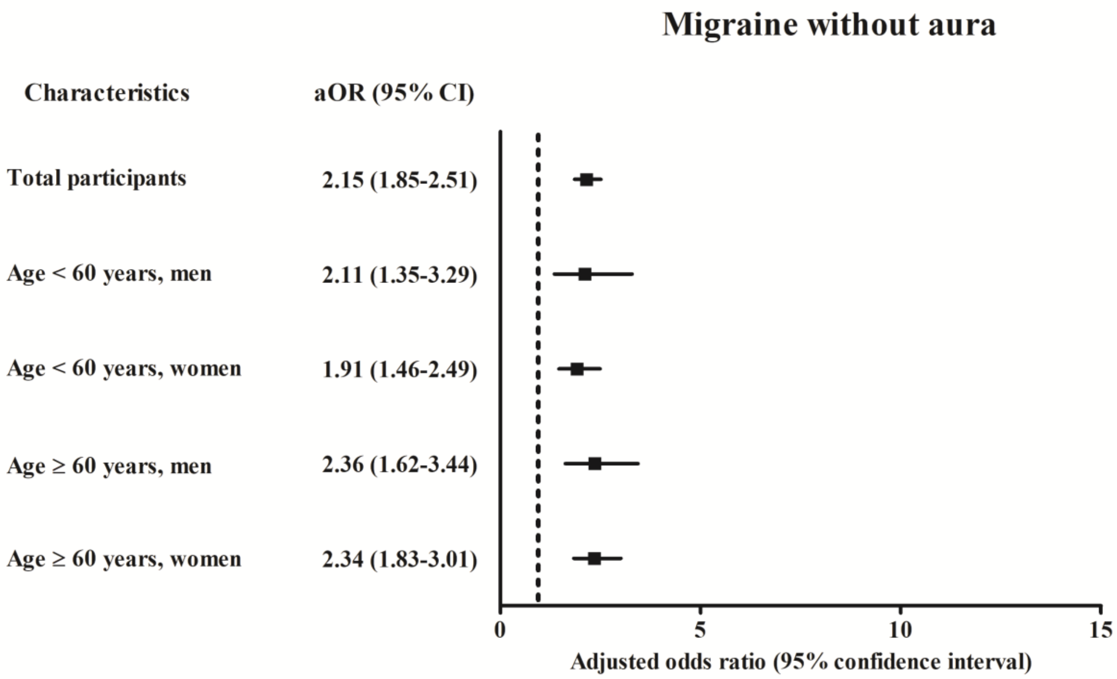

Figure 2. Subgroup analyses of crude and adjusted hazard ratios (95% confidence interval) for migraine without aura in temporomandibular disorder and control groups.

Table 1. Subgroup analyses of crude and adjusted hazard ratios (95% confidence interval) for migraine with aura in temporomandibular disorder and control groups.

Characteristics	Hazard ratios for migraine with aura			
	**Crude†**	***p*-value**	Adjusted†‡	*p*-value
Total participants (*n* = 19420)				
TMD	1.29 (0.63-2.63)	0.485	1.33 (0.65-2.74)	0.434
Control	1		1	
Age < 60 years old, men (*n* = 4,040)				
TMD	1.02 (0.11-9.17)	0.983	1.23 (0.11-14.03)	0.868
Control	1.00		1.00	
Age < 60 years old, women (*n* = 4,890)				
TMD	1.61 (0.63-4.16)	0.322	1.53 (0.58-4.01)	0.389
Control	1.00		1.00	
Age ≥ 60 years old, men (*n* = 4,725)				
TMD	0.95 (0.11-8.52)	0.964	0.94 (0.08-10.49)	0.959
Control	1.00		1.00	
Age ≥ 60 years old, women (*n* = 5,765)				
TMD	0.99 (0.21-4.67)	0.992	0.83 (0.17-4.03)	0.817
Control	1.00		1.00	

CCI, Charlson Comorbidity Index; TMD, temporomandibular disorder; * Stratified Cox proportional hazard regression model, significance at $p < 0.05$; † Models were stratified by age, sex, income, and region of residence. ‡ The model was adjusted for obesity, smoking, alcohol consumption, systolic blood pressure, diastolic blood pressure, fasting blood glucose, total cholesterol, and CCI scores.

Table 2. Subgroup analyses of crude and adjusted hazard ratios (95% confidence interval) for migraine without aura in temporomandibular disorder and control groups.

Characteristics	Hazard ratios for migraine without aura			
	**Crude†**	***p*-value**	**Adjusted†‡**	***p*-value**
Total participants (n = 19,420)				
TMD	2.18 (1.87-2.53)	<0.001*	2.15 (1.85-2.51)	<0.001*
Control	1		1	
Age < 60 years old, men (n = 4,040)				
TMD	2.14 (1.38-3.33)	<0.001*	2.11 (1.35-3.29)	0.001*
Control	1.00		1.00	
Age < 60 years old, women (n = 4,890)				
TMD	1.94 (1.49-2.53)	<0.001*	1.91 (1.46-2.49)	<0.001*
Control	1.00		1.00	
Age ≥ 60 years old, men (n = 4,725)				
TMD	2.30 (1.59-3.34)	<0.001*	2.36 (1.62-3.44)	<0.001*
Control	1.00		1.00	
Age ≥ 60 years old, women (n = 5,765)				
TMD	2.36 (1.84-3.02)	<0.001*	2.34 (1.83-3.01)	<0.001*
Control	1.00		1.00	

CCI, Charlson Comorbidity Index; TMD, temporomandibular disorder; * Stratified Cox proportional hazard regression model, significance at $P < 0.05$; † Models were stratified by age, sex, income, and region of residence. ‡ The model was adjusted for obesity, smoking, alcohol consumption, systolic blood pressure, diastolic blood pressure, fasting blood glucose, total cholesterol, and CCI scores.
